# Differential Modulation of Mouse Heart Gene Expression by Infection With Two *Trypanosoma cruzi* Strains: A Transcriptome Analysis

**DOI:** 10.3389/fgene.2020.01031

**Published:** 2020-09-03

**Authors:** Tiago Bruno Rezende de Castro, Maria Cecilia Campos Canesso, Mariana Boroni, Daniela Ferreira Chame, Daniela de Laet Souza, Nayara Evelin de Toledo, Eric Birelli Tahara, Sergio Danilo Pena, Carlos Renato Machado, Egler Chiari, Andrea Macedo, Gloria Regina Franco

**Affiliations:** ^1^Departamento de Bioquímica e Imunologia, Universidade Federal de Minas Gerais, UFMG, Belo Horizonte, Brazil; ^2^Laboratório de Bioinformática e Biologia Computacional, Centro de Pesquisas, Instituto Nacional de Câncer, Rio de Janeiro, Brazil; ^3^Departamento de Parasitologia, Universidade Federal de Minas Gerais, UFMG, Belo Horizonte, Brazil

**Keywords:** *Trypanosoma cruzi*, host-parasite interaction, heart transcriptome, immune system, differential gene expression

## Abstract

The protozoan *Trypanosoma cruzi* (*T. cruzi*) is a well-adapted parasite to mammalian hosts and the pathogen of Chagas disease in humans. As both host and *T. cruzi* are highly genetically diverse, many variables come into play during infection, making disease outcomes difficult to predict. One important challenge in the field of Chagas disease research is determining the main factors leading to parasite establishment in the chronic stage in some organs, mainly the heart and/or digestive system. Our group previously showed that distinct strains of *T. cruzi* (JG and Col1.7G2) acquired differential tissue distribution in the chronic stage in dually infected BALB/c mice. To investigate changes in the host triggered by the two distinct *T. cruzi* strains, we assessed the gene expression profiles of BALB/c mouse hearts infected with either JG, Col1.7G2 or an equivalent mixture of both parasites during the initial phase of infection. This study demonstrates the clear differences in modulation of host gene expression by both parasites. Col1.7G2 strongly activated Th1-polarized immune signature genes, whereas JG caused only minor activation of the host immune response. Moreover, JG strongly reduced the expression of genes encoding ribosomal proteins and mitochondrial proteins related to the electron transport chain. Interestingly, the evaluation of gene expression in mice inoculated with a mixture of the parasites produced expression profiles with both up- and downregulated genes, indicating the coexistence of both parasite strains in the heart during the acute phase. This study suggests that different strains of *T. cruzi* may be distinguished by their efficiency in activating the immune system, modulating host energy metabolism and reactive oxygen species production and decreasing protein synthesis during early infection, which may be crucial for parasite persistence in specific organs.

## Author Summary

The causative agent of Chagas disease, *Trypanosoma cruzi*, retains high genetic diversity, and its populations vary greatly in different geographic locations. *T. cruzi* mammalian hosts, including humans, also have high genetic variation, making it difficult to predict the disease outcome. Accordingly, this variability must be taken into account in studies aiming to determine the effect of polyparasitism on drug trials, vaccine studies, diagnosis or basic research. Therefore, there is a growing need to consider the interaction between the pathogen and the host immune system in mixed infections. In the present work, we show an in-depth analysis of gene expression in hearts from BALB/c mice infected with either Col1.7G2 or JG alone or a mixture of both strains. Col1.7G2 induced a higher Th1 inflammatory response, while JG produced a weaker activation of immune response genes. Furthermore, JG-infected mice showed a notable reduction in the expression of genes responsible for mitochondrial oxidative phosphorylation and protein synthesis. Interestingly, the mixture-infected group displayed changes in gene expression caused by both strains. Overall, we provided new insights into the host-pathogen interaction in the context of single and dual infection by showing the remarkable differences in the modulation of host gene expression by the two *T. cruzi* strains.

## Introduction

Chagas disease (CD) is a parasitic illness caused by the kinetoplastid protozoan *Trypanosoma cruzi*. Six to seven million people are estimated to be infected by this disease, which mostly affects poor communities in rural areas of Latin America (World Health Organization [WHO], 2020). Despite being a prototypical neglected tropical disease, CD has recently gained attention in non-endemic areas due to the increasing emigration of affected people from endemic to non-endemic countries, and new cases have resulted mainly from blood transfusion, organ transplantation and congenital transmission (Coura and Viñas, 2010; Coura, 2015; Meymandi et al., 2017). To date, six discrete typing units (DTUs I-VI) have been described for *T. cruzi* according to a series of genetic markers, such as rDNA 24Sα, mini-exon and mitochondrial polymorphisms (Souto et al., 1996; De Freitas et al., 2006; Burgos et al., 2007; Zingales et al., 2011), and a seventh has been postulated (Tcbat) (Marcili et al., 2009). This broad genetic diversity makes *T. cruzi* a highly complex organism and plays an essential role in its differential tropism in host tissues, which culminates in the diverse clinical manifestations observed in chronic patients and experimental models of CD (Vago et al., 1996, 2000; Andrade et al., 1999, 2002). Notably, the term tropism has been used with different meanings by many authors in the scientific literature (Gussin, 1963; Husson, 1968; McFadden et al., 2009), and here, we define tissue tropism as the ability of a particular pathogen to infect and persist within an organ or a set of organs (McCall et al., 2016).

Elucidating the molecular mechanisms dictating the interaction between the pathogen and its host is crucial for understanding disease progression and the development of new treatments. Previously, Andrade et al. showed that after inoculating BALB/c mice with a mixture of different strains of *T. cruzi*, JG (*T. cruzi* II) and Col1.7G2 (*T. cruzi* I), the two parasite strains were not evenly distributed among different tissues in the chronic phase of the disease. JG was primarily found in the heart, while Col1.7G2 was encountered in the rectum of the animals (Vago et al., 2000; Andrade et al., 2002). Curiously, mice infected with only one strain did not exhibit this pronounced tissue tropism. In addition, the use of different mouse lineages, such as C57BL/6J and SWISS, was unable to reproduce the aforementioned tissue tropism, indicating the role of the host in the different behaviors of the *T. cruzi* strains. Furthermore, such tropism was also detected in human patients during the chronic phase of CD, as distinct *T. cruzi* DTUs were established in different organs, leading to the development of the “clonal-histotropic model” hypothesis (Vago et al., 1996, 2000; Macedo and Pena, 1998).

Recently, extensive research has improved the understanding of the immunological and molecular interactions between *T. cruzi* and its mammalian hosts. An efficient and non-exaggerated immune response is crucial to pathogen clearance, without much damage to the host tissue (Holscher et al., 2000). The innate immune system provides the first line of defense to initiate an effective response against a parasite via pattern recognition receptors (PRRs), of which the Toll-like receptors (TLRs) are the best known (Takeuchi and Akira, 2010). Among the most well-studied TLRs in the context of CD are *Tlr-2* and *Tlr-9* (Bartholomeu et al., 2008). Glycosylphosphatidylinositol (GPI)-anchored mucin-like glycoproteins (tGPI-mucins), which are widely present on the parasite surface, and unmethylated CpG DNA sequences are the primary immunostimulatory ligands of these TLRs (De Souza et al., 2010). Their interaction has been shown to trigger the release of IL-12 and TNF by dendritic cells and macrophages, which are pivotal for host resistance at the beginning of the acute phase of CD (Bafica et al., 2006; Gravina et al., 2013). Moreover, the establishment of a Th1 response, which is dependent on the release of IFN-γ, has been extensively studied and is essential for parasite clearance and host survival (Aliberti et al., 1996; Bartholomeu et al., 2008). However, there is a lack of comparative studies of the differences in the host response against distinct strains of *T. cruzi* and their role in differential parasite tissue preferences. Experiments in mice and rats comparing JG (*T. cruzi* II) and CL-Brenner (*T. cruzi* VI) have shown remarkable differences in the systemic production of inflammatory cytokines and inflammatory cells (Franco et al., 2003; Bartholomeu et al., 2008). Although much progress has been made in understanding the complexity of the tissue preferences of the parasite, the actual changes in host gene expression during infection with distinct *T. cruzi* strains and mixtures have not yet been investigated.

In the present work, we report a thorough gene expression analysis of BALB/c hearts during the acute stage of infection by different strains of *T. cruzi* and a mixture of strains. To our knowledge, our work depicts the first deep RNA-Seq data comparing distinct effects of *T. cruzi* strains, *in vivo*. Our data reveal that JG-infected mice exhibit less pronounced induction of both innate and adaptive immune response genes compared to Col1.7G2-infected mice. Moreover, biological processes such as translation and mitochondrial oxidative phosphorylation are strongly downregulated in JG-infected mice, indicating strong modulation of cellular metabolism caused by JG infection. Remarkably, the mixture-infected animals showed both profiles simultaneously. Our data demonstrate the complexity of host-pathogen interactions in the context of experimental CD and how distinct *T. cruzi* strains differentially affect gene expression in mouse hearts.

## Materials and Methods

### Ethics Statement

All animal procedures described in this study complied with the standards given in the Guide for the Care and Use of Laboratory Animals and were conducted under conditions approved by the local animal ethics committee (National Research Council (US) Committee for the Update of the Guide for the Care and Use of Laboratory Animals, 2011). The Institutional Committee for Animal Ethics of UFMG (CEUA-UFMG, license 64/12) approved all experimental procedures used in this study. The *in vivo* experiments were conducted during the valid timeline of the license. Both non-infected and infected animals were kept in plastic boxes with food and water that was available *ad libitum* with appropriate technical management in cages that were properly identified and sealed in a 12 h light-dark cycle environment. All animal procedures were performed under anesthesia using a mixture of ketamine (50 mg/kg) and xylazine (10 mg/kg). In the end of each experiment, infected animal carcasses were properly autoclaved to eliminate all living parasites before final disposal.

The *T. cruzi* populations used in this study, Col1.7G2 and JG, belong to lineages TCI and TCII, respectively. The JG strain was originally isolated in 1995 by Professor Eliane Lages-Silva (Universiade Federal do Triangulo Mineiro, Brazil) from a chronic patient with megaesophagus. The Col1.7G2 strain is a clone from the Colombian strain, which was originally isolated by Federici in 1964 from a chronic patient with cardiac disorder (Federici et al., 1964). Both JG and Col1.7G2 were formerly characterized as monoclonal populations, through the analysis of the eight microsatellite loci according to previously described methodology (Oliveira et al., 1998). Both strains belong to the *T. cruzi* collection at the Laboratory of Chagas Disease (UFMG), which is coordinated by Professor Egler Chiari. Infective trypomastigote forms were thawed after liquid nitrogen preservation, and the parasites were injected into SWISS mice for population expansion, after which they were diluted to 50 parasites/100 μl of sterile PBS for further use in the BALB/c infections.

### Experimental Infection

**FIGURE 1:**
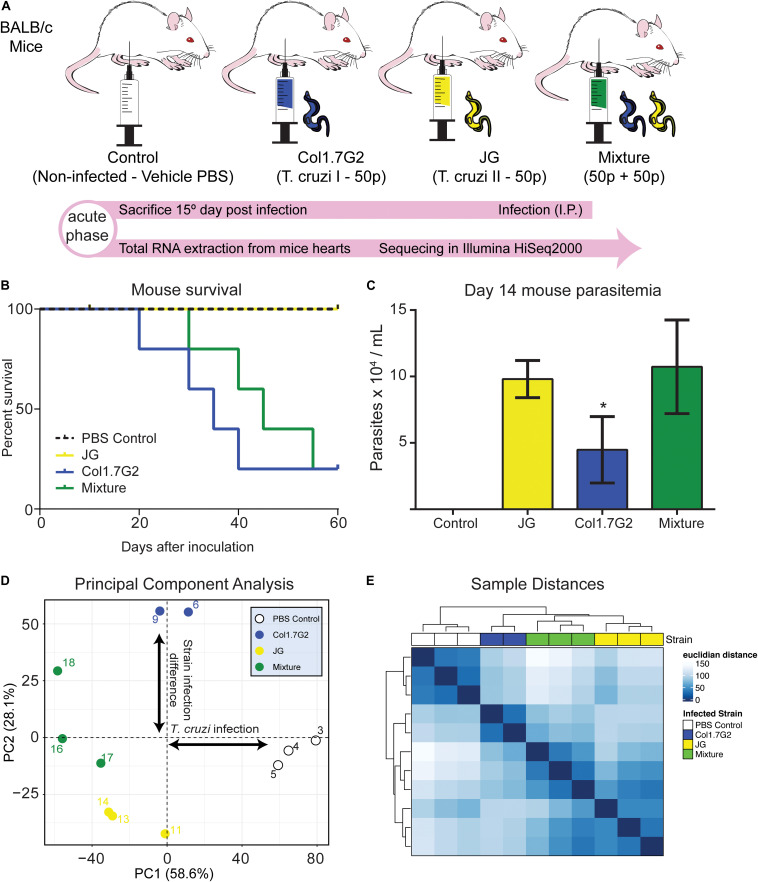
Differential gene expression profile of mouse hearts infected by two *T. cruzi* strains. **(A)** BALB/c mice were divided into one non-infected control and three experimental groups, infected with Col1.7G2, JG, or a mixture of the strains with equivalent quantities of both strains. After 15 days, during the acute phase, the mice were euthanized, and the hearts were removed for total RNA extraction and sequencing with an Illumina HiSeq2000. **(B)** Survival curve of mice infected by Col1.7G2, JG and its mixture 60 days. **(C)** Mouse parasitemia 14 days after infection. Data represent the mean ± SEM, *n* = 5 mice/group. *p < 0.05 when comparing the Col1.7G2 group to the JG and mixture groups based on the Kruskal-Wallis test followed by a *post hoc* Dunn test. **(D)** To determine the overall expression profile in each mouse sample, the controls (blank circles), Col1.7G2-infected mice (blue circles), JG-infected mice (yellow circles) and mixture-infected mice (green circles) were analyzed by performing principal component analysis (PCA). The first two principal components (PC1 and PC2) are plotted according to the rlog-transformed data of the read counts for each gene. **(E)** Hierarchical clustering of the rlog-transformed data matrix is shown as a heatmap with the Euclidian distances between the samples.

Inbred 6–8-week-old male BALB/c mice were obtained from the Centro de Bioterismo (CEBIO/ICB, Belo Horizonte, Brazil) and housed in our local animal facility under the same conditions. The mice were randomly divided into four groups of 5 individuals each. The non-infected controls were injected intraperitoneally (i.p.) with phosphate-buffered saline (PBS-vehicle), while the three experimental groups were injected (i.p.) with 50 JG (*T. cruzi* II) trypomastigotes, 50 Col1.7G2 (*T. cruzi* I) trypomastigotes or an artificial mixture of both strains (50 + 50 parasites) (Figure 1A). After infection, the mice were caged according to group. Blood parasitemia was verified by collecting 5 μL of blood from the animal tail and parasites were counted in optical microscope on the 14th day post infection. On the 15th day, the mice were anesthetized, and their hearts were collected, rapidly washed in sterile PBS and immediately frozen in liquid nitrogen.

### RNA Extraction, Quality Assessment, and Sequencing

The frozen hearts were kept in liquid nitrogen and thoroughly pulverized in their entirety into a powder using a sterile porcelain crucible. The total RNA used for sequencing was extracted using the Trizol reagent (Life Technologies) according to the manufacturer’s protocol and precipitated with isopropanol. The quality and integrity of the samples were verified by capillary gel electrophoresis on a Bioanalyzer 2100 (Agilent Technologies). To access the RNA grade for sequencing, the RNA integrity number (RIN) score was calculated for each sample with Bioanalyzer software. Samples with RIN scores greater than 6 were considered for downstream analysis (Supplementary Table S1; Fleige and Pfaffl, 2006; Schroeder et al., 2006).

The cDNA libraries were prepared and sequenced at the Beijing Genomics Institute (BGI – Shenzhen, China). Samples were submitted for sequencing accordingly to the minimal quality requirements determined by BGI. In brief, polyadenylated RNA was purified from total RNA, converted to cDNA using random hexamer primers, sheared, and size-selected for fragments ∼200 bp in length using the Illumina TruSeq RNA Sample Preparation Kit v2. In order to obtain approximately 10 million paired-end reads per sample within the 120 million read package offered by BGI, we chose the 3 higher quality scored samples per group for downstream procedures. Sequencing was performed on the Illumina HiSeq 2000 (Illumina, CA) platform and generated approximately 12 million paired-end reads (Supplementary Table S1), which were 90 nucleotides in length, for each sample.

### Processing of Raw Sequencing Reads

The raw reads were first checked for quality using FastQC^1^ software (Babraham Bioinformatics, Cambridge, United Kingdom) (Andrews, 2010). Since all samples had high quality scores, no sequence trimming was performed (Supplementary Figure S1). To detect parasite derived sequences, we aligned the reads to the most recent *T. cruzi* genome assembly, TriTrypDB-43 (TcruziCLBrenerEsmeraldo-like, TcII) (El-Sayed et al., 2005) and eliminated those reads derived from mouse mRNA. We also used a *de novo* approach with Trinity (Haas et al., 2013) to assemble the parasite sequences into full RNA transcripts. Subsequently, we used NCBI BLAST to annotate and then, highly conserved transcripts between mouse and *T. cruzi* was removed. Finally, we realigned and quantified all samples against the assembled *T. cruzi* transcriptome (Supplementary Figures S1, S2). The samples number 8 and 11 had low amount of parasite mapped reads. A noise originated from conserved sequences is also observed in non-infected mice.

### Differential Expression

Principal component analysis (PCA) was performed by using the prcomp package and visualized with factoextra^2^ and (R Core Team, 2020) factominer^3^ packages (Lê et al., 2008). Initially, PCA suggested that sample 8 of the group Col1.7G2 had gene expression profile very similar to non-infected animals (Supplementary Figure S3). Thus, together with the low number of parasite reads (Supplementary Figure S2), we decided to exclude it from subsequent analysis as it suggested that this sample was not able to develop a fruitful infection, compared to the other samples. Gene expression was assessed by using the raw FASTQ files and the mouse reference transcriptome M21 (Frankish et al., 2019), Gencode – GRCm38.p6, as input for Kallisto software^4^ (Bray et al., 2016). The obtained normalized transcript abundances, measured as Transcripts Per Kilobase Million (TPM), were processed by the R package Sleuth for differential analysis^5^ (Pimentel et al., 2017). The analyses were conducted at the gene level, and a likelihood ratio test was applied to determine the gene expression differences among all groups. When testing between group pairs, the Wald test was used to determine the differentially expressed genes (Yu et al., 2017). We considered as significant for downstream studies genes with log2-fold changes > 1 or <−1 and a false discovery rate of 0.01 as significant. Lastly, we used the elbow method to determine the ideal number of clusters by testing 1–15 possible k-values. The clustered genes were then plotted in a heatmap using the ComplexHeatmap^6^ package (Gu et al., 2016).

### Analysis of Non-significant Gene Sharing

We sought to study the distribution of exclusive DEGs in all experimental groups to verify the level of real sharing or exclusiveness. The list of exclusive DEGs (see Supplementary Table S3) in JG-infected (168), Col1.7G2-infected (274) and Mixture-infected groups (906) were searched in the other experimental groups for its non-significant *q*-values and log2 fold change. The four major comparisons were CiJ (Exclusive significant Col1.7G2 DEGs in the non-significant JG genes), JiC (Exclusive significant JG DEGs in the non-significant Col1.7G2 genes), MiC (Exclusive significant Mixture DEGs in the non-significant Col1.7G2 genes), MiJ (Exclusive significant Mixture DEGs in the non-significant JG genes). Later, we performed a Kruskal-Wallis test with a *post hoc* Dunn’s test to determine if the distribution was significantly different.

### Enrichment Analysis

We conducted the enrichment analysis using the R package with mouse genome-wide annotations^7^ (Carlson, 2017). The functional enrichment for biological processes (based on Fisher’s exact test) was calculated with the Bioconductor package topGO^8^ (Alexa and Rahnenfuhrer, 2016). We analyzed the upregulated and downregulated genes separately, as described previously (Hong et al., 2013).

### Functional Network Analysis

The networks of enriched functional terms were constructed with the ClueGO plug-in^9^ using Cytoscape software (Shannon et al., 2003; Bindea et al., 2009). The lists of DEGs were analyzed using the EBI-QuickGO mouse annotation for biological processes. Pathways with *p* < 0.01 were considered statistically significant after Bonferroni step-down correction.

### Quantitative Real-Time PCR (qPCR)

Total RNA from mouse hearts was extracted using TRIzol (Life Technologies, CA, United States) and treated with TURBO DNase (Thermo Fisher Scientific, MA, United States). cDNA was then produced with the Superscript II first strand synthesis kit (Invitrogen, CA, United States). The PCR experiments were performed with a 7900HT Fast Real-Time PCR System (Applied Biosystems, CA, United States) using the PowerUp SYBR Green Master Mix (Thermo Fisher Scientific, MA, United States). The tested genes and primer sequences are provided in Supplementary Table S8. To evaluate the expression levels of tested genes in each sample, qPCR measurements were normalized to the expression of *Gapdh* and the fold change values were calculated with the 2^–ΔΔ*CT*^ method.

### Data Availability

All raw and processed sequencing data are available in the GEO (Gene Expression Omnibus) database under the accession code GSE132132.

## Results

### JG and Col1.7G2 Infections Exhibit Distinct Profiles

To follow the course of infection by JG and Col1.7G2 strains, we inoculated BALB/c mice with a single strain or a mixture of both strains (Figure 1A) and evaluated the survival of the animals until the early chronic phase of the infection was reached (Figure 1B). We observed that during the early acute phase, Col1.7G2-infected mice exhibited lower parasitemia when compared to the JG- and mixture-infected mice (Figure 1C). To assess transcriptional changes in the mouse hearts in response to infection with Col1.7G2, JG, and the mixture, we performed total mRNA sequencing from whole mouse hearts on the 15th day of infection by high-throughput sequencing. The general expression profile of each sample was determined by PCA (Figure 1D). The modulation of host gene expression in response to parasitism was determined according to the first principal component. We could also determine the significant differences in gene expression between the groups infected by these two *T. cruzi* strains according to the second principal component. Samples from mice infected with both strains showed a gene expression profile that was intermediate compared to those found in mice infected with each strain individually. This profile was better represented by the Euclidean distance, which shows the extent of the sample-sample differences (Figure 1E). We could also verify that the mixture-infected group shows similarities with both strain-infected groups.

### JG and Col1.7G2 Infections Differentially Modulate Heart Gene Expression

**FIGURE 2:**
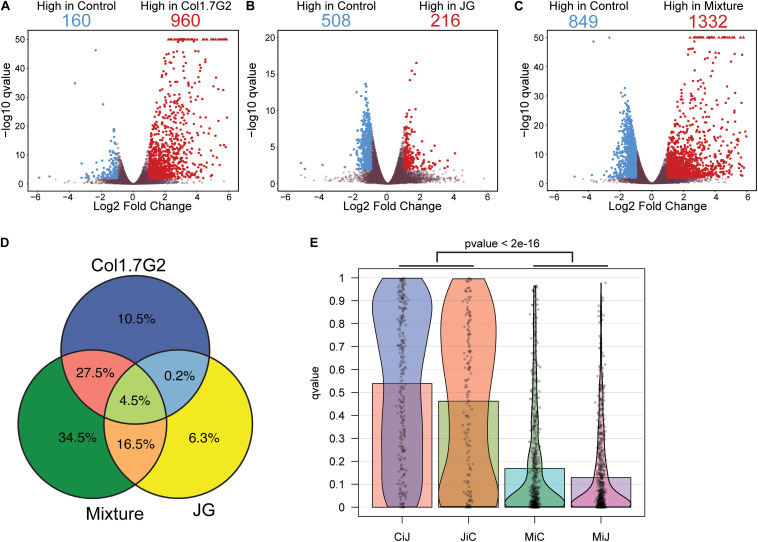
Comparative analysis of mouse gene expression after exposure to single and mixed *T. cruzi* infections. Volcano plot of **(A)** Col1. 7G2-, **(B)** JG- and **(C)** mixture-infected groups showing the log2 fold changes for each detected gene on the x-axis along with the -log10 adjusted *p*-values after comparison with non-infected controls on the *y*-axis. Blue colored dots represent significant genes with a log2 fold change < -1 while red colored dots represent significant genes with a log2 fold change greater than 1. Gray dots represent genes that were not considered differentially expressed **(D)**. The Venn diagram shows the proportions of shared and exclusive genes in each group (see Supplementary Table S3 for gene names). **(E)** Distribution of the adjusted *p*-values (qvalue) of exclusive DEGs from Col1.7G2 or JG-infected groups present in the non-significant gene list of JG (CiJ) and Col1.7G2-infected groups (JiC) respectively, as well as the distribution of exclusive DEGs from the Mixture found in the non-significant lists of Col1.7G2 (MiC) or JG (MiJ). The *p*-value < 2e-16 represent the comparison of MiC or MiJ against CiJ or JiC using the Wilcoxon rank sum test.

To evaluate the overall gene expression changes during *T. cruzi* infection compared to those in the uninfected controls, we performed pairwise comparisons using the Wald test. We found that approximately 14,400 genes with TPM > 0 were expressed in mouse hearts in all experimental groups (Supplementary Table S2). The distribution of the differentially expressed genes (DEGs) in each group is represented by volcano plots (Figures 2A–C). It is worth noting that there were remarkable differences in the fold changes of the DEGs among the groups. Col1.7G2-infected animals exhibited more upregulated (960 in Col1.7G2 and 216 in JG) and fewer downregulated DEGs (160 in Col1.7G2 and 508 in JG) relative to the control. Notably, mouse hearts from the mixed infection group had a higher number of downregulated DEGs (849) and a higher number of upregulated DEGs (1332) relative to the control group when compared to both single-infected groups. We next evaluated genes that were shared or exclusively expressed in each group. Thus, we constructed a Venn diagram with all 2635 genes that were detected in the pairwise comparisons (Figure 2D and Supplementary Table S3). The DEGs shared by all groups comprised only 4.5% of the total, and these genes may be associated with *T cruzi* infection. The Col1.7G2 and mixture groups shared 27.5% of their DEGs, and the JG and the mixture groups shared 16.5%. However, the JG and Col1.7G2 groups did not share DEGs other than the ones that were also present in the mixture group, revealing that the two kinds of infection may modulate specific classes of genes. It is worth noting that 10.5% of the DEGs were exclusive to Col1.7G2 infection, and 6.3% of the DEGs were exclusive to the group infected with JG. The mixture had 34.5% of the exclusive DEGs, suggesting the uniqueness of this kind of infection. Hence, we evaluated the distribution of the false discovery rate (FDR or *q*-values) values of the exclusive DEGs from the Col1.7G2, JG and mixture-infected groups that were not considered DEGs in the other groups (Figure 2E). Notably, we observed an accumulation of mixture-exclusive DEGs that were close to the threshold of also being considered DEGs (FDR < 0.01) in the JG or Col1.7G2 group, but this was not observed for DEGs exclusive to the JG group that were present in the Col1.7G2 group and vice versa; this demonstrated that these two groups are distinct and that most of the exclusive DEGs in the mixture group were actually also present in the Col1.7G2 or JG group. Since our study sequenced mRNA from infected whole heart tissue, we were also able to detect sequence reads derived from the parasites (Supplementary Figure S2). Due to the higher amount of host mRNA in comparison with pathogen mRNA, we only detected a total of 636 parasite transcripts. The lack of sequenced genomes for the JG and Col1.7G2 strains makes it unfeasible to confirm the strain origin. However, it serves as an indirect indicative of the parasite presence in the tissue.

### Analyses of the Gene Expression Profiles and Enriched Biological Processes Reveal the Global Signatures of the Early Infection of JG and Col1.7G2 in the Heart

**FIGURE 3:**
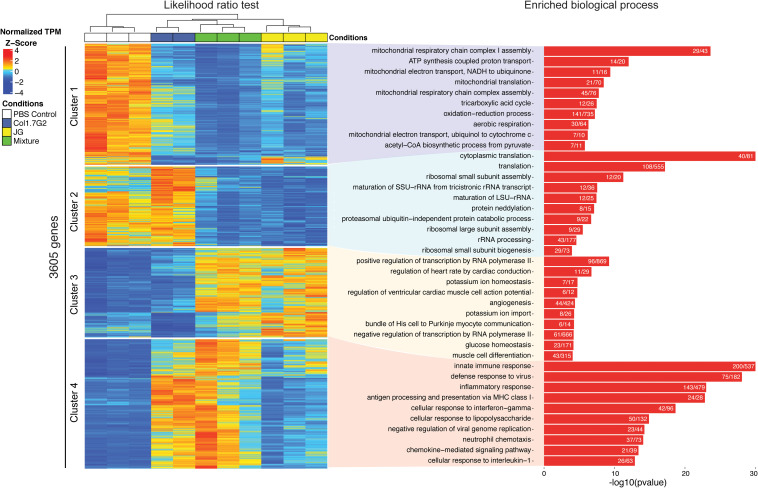
Analysis of enriched Gene Ontology categories represented by the differentially expressed genes. All genes from the Col1.7G2, JG, and mixture groups were analyzed in accordance with their Gene Ontology terms using the topGO package for R. The encountered GO terms derived from the up- and downregulated genes are shown in red and blue, respectively. The bars represent the -log10 values of the Fisher elim *p*-values (*p* < 0.01). Histogram values represent the number of differentially expressed genes based on the number of expressed genes in the background.

To reveal the differences between the gene expression profiles of each group, we used a likelihood ratio test to generate an unbiased list of DEGs. In total, we found 3605 genes with a *q*-value (*p*-value-adjusted) lower than 0.01 (Supplementary Table S4 and Figure 3 left). The heatmap shows four major clusters of genes as determined by the elbow method (Supplementary Tables S5, S6). Cluster 1 mainly consists of genes downregulated in the infected groups compared with the non-infected control group, and cluster 2 exhibits genes that are downregulated in the JG- and mixture-infected groups compared with the control and Col1.7G2-infected groups. Clusters 3 and 4 show upregulated genes that the mixture-infected group shares with the JG- and Col1.7G2-infected groups, respectively. Next, to investigate the enriched biological processes represented by each cluster of genes, we utilized the topGO package (Supplementary Tables S6, S7 and Figure 3 right). Remarkably, cluster 1 shows high enrichment in processes related to mitochondrial metabolism, such as ATP synthesis (14 significant/20 total annotated), tricarboxylic acid cycle (12 significant/26 total annotated) and oxidation-reduction (141 significant/735 total annotated). It is worth noting that the downregulation of these genes is greater in the mixture- and JG-infected animals than in the Col1.7G2-infected animals. Surprisingly, cluster 2 exhibits the strong downregulation of genes related to protein translation (108 significant/555 total annotated), protein metabolism, such as neddylation (8 significant/15 total annotated), and ribosomal subunit processing (29 significant/73 total annotated) when comparing the JG- and mixture-infected groups but not the Col1.7G2-infected and control groups. Cluster 3 comprises genes upregulated only in the JG and the mixture-infected samples and is enriched in many processes associated with heart functioning, such as angiogenesis (44 significant/424 total annotated), muscle cell differentiation (43 significant/315 total annotated), and the regulation of heart rate by cardiac conduction (11 significant/29 total annotated). Finally, cluster 4 mainly exhibits genes involved in immune system activation and was strongly upregulated in Col1.7G2- and mixture-infected animals. It is worth mentioning that processes such as neutrophil chemotaxis (37 significant/73 total annotated), antigen presentation (24 significant/28 total annotated) and innate immune responses (200 significant/537 total annotated) were the main activated processes.

**FIGURE 4:**
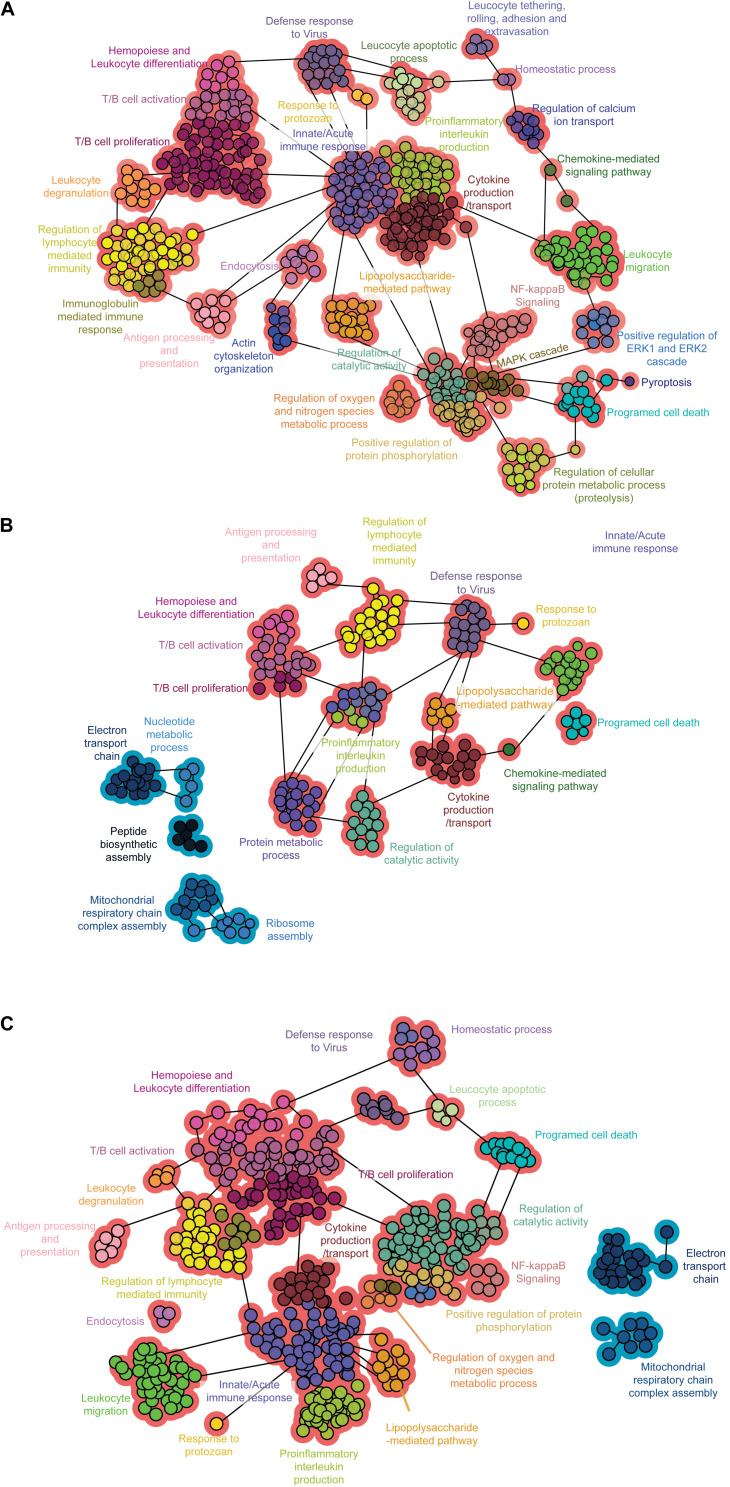
Functional networks of enriched biological processes. Network of the biological processes in the **(A)** Col1. 7G2-, **(B)** JG-, and **(C)** mixture-infected groups. Functional groups with more than 90% upregulated or downregulated genes are shown in red or blue, respectively. The data represent only gene clusters with *p* < 0.001 after Bonferroni step-down correction.

To visualize the interactions among the enriched biological processes representing DEGs, we constructed functional networks for each infected group in comparison with the control using the Cytoscape plugin ClueGO. The genes activated by Col1.7G2 infection were related to inflammatory pathways such as innate and adaptive immune responses, especially cytokine production (Figure 4A). The JG-infected samples showed fewer immune response-mediated pathways when compared to the Col1.7G2-infected group. However, it is evident that mitochondrial processes and the electron transport chain are downregulated as a result of JG infection (Figure 4B). It is worth noting that the Mixture-infected group displays networks of upregulated immune response genes and the downregulation of mitochondrial-related genes, which reflected the presence of Col1.7G2- and JG-infected cells (Figure 4C).

**FIGURE 5:**
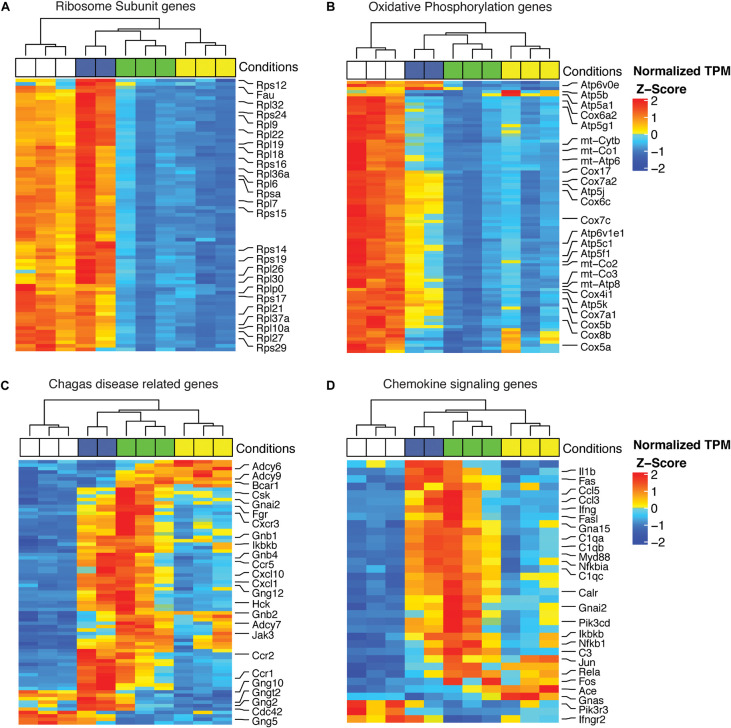
Heatmap of DEGs involved in **(A)** ribosomal subunits, **(B)** the oxidative phosphorylation pathway, **(C)** Chagas disease-related genes and **(D)** chemokine signaling. The data represent the z-scores of the normalized read counts (log2 scale) for each gene. The base mean track shows the average gene expression in the samples.

To visualize the effects of infection with Col1.7G2, JG and the mixture of both strains on the modulation of genes that play specific roles in protein synthesis, mitochondrial processes and inflammation, we generated heatmaps by using the KEGG database. The complete expression data for all genes with expression bigger than Log2_*T*__*PM*_ > 0, and experimental groups are presented in the Supplementary Material (Supplementary Table S2). The large and small ribosomal subunits were significantly downregulated in the JG-infected group but not in the Col1.7G2-infected group (Figure 5A). Oxidative phosphorylation was strongly downregulated in JG- and mixture-infected animals when compared to Col1.7G2-infected animals (Figure 5B). The KEGG-identified genes within the set of Chagas disease-related genes (inflammatory proteins) were predominantly upregulated in Col1.7G2- and mixture-infected animals (Figure 5C). A similar profile was observed for the set of chemokine signaling genes (Figure 5D).

### Gene Expression Validation by qPCR

**FIGURE 6:**
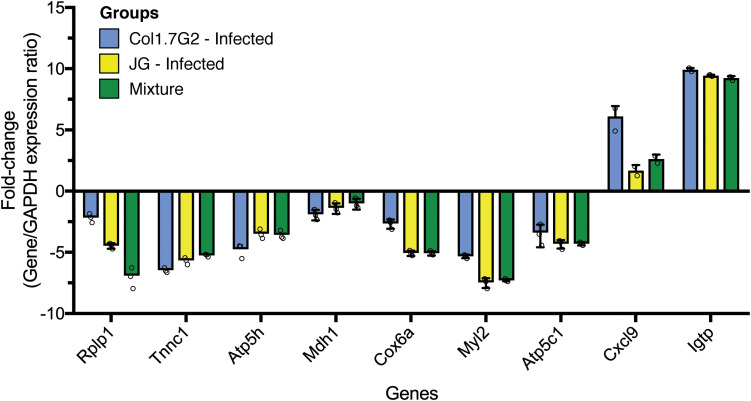
Quantitative real-time RT-PCR validation of differentially expressed genes. Expression levels of tested genes were normalized to the expression of Gapdh and the fold change values were calculated with the 2^–ΔΔ*CT*^ method. All PCRs were performed with three technical replicates.

To confirm the expression data obtained from RNA-Seq, we performed qPCR for validation (Figure 6). The quantitative PCR data show that the fold changes of most of the tested genes corroborated the reported RNA-Seq expression data. Highly upregulated genes in the RNA-Seq analysis, such as *Cxcl9* and *Igtp*, were also upregulated according to qPCR when compared to *Gapdh* expression. Genes encoding proteins operating in the mitochondria were shown to be downregulated by both methods. The data represent one sample from each group with three technical replicates.

## Discussion

One critical aspect of the distinct *T. cruzi* strains is their high genetic variability and differential parasitism when infecting a mammalian host. Our research group has intensively studied the Col1.7G2 (*T. cruzi I)* and JG (*T. cruzi II*) strains during the course of infection in different mouse lineages and tissue tropism (Andrade et al., 1999, 2002, 2010). While the JG strain rarely causes death in BALB/c mice, Col1.7G2 results in high virulence and kills animals during the early stages of infection. However, the observed virulence does not appear to be caused by parasite burden, since JG-infected mice display higher parasitemia than Col1.7G2-infected mice in the acute phase. We have shown that the mixture-infected animals presented high parasitemia, as seen in JG and high mortality rate, as seen in Col1.7G2-infected mice. Previous studies that evaluated more closely the impact of mixed infections obtained strain-dependent results. Campos et al., observed that BALB/c mice infected by two *T. cruzi* I strains (AQ1-7 and MUTUM) showed undetectable parasitemia that overly increased when applied as a mixture (Sales-Campos et al., 2015). Perez et al. showed that mixed infections did not increase the mortality rate compared to that of single infections, although higher parasitemia was observed (Perez et al., 2018). Rodrigues et al. observed diminished mortality in animals infected with a mixture of the JG and CL Brenner strains when compared to that in animals with single infections (Rodrigues et al., 2010).

Since it remains an open question as to whether mixed infection leads to the exacerbation of the pathogenic effects of single strains (synergistic effect) or merely the sum of the impacts of both strains (Rodrigues et al., 2010; Magalhães et al., 2015; Perez et al., 2018), we evaluated DEGs shared among the infected samples and the numbers of exclusive and shared genes in the mixture-infected animals. Although we detected a high percentage of exclusive genes in the mixture (close to 35%), a more thorough evaluation of the *q*-value distribution of these genes in the other groups led to the conclusion that the effects of mixed infection are much more similar to the sum of the effects of each single infection than previously thought, due to the large number of gene enrichments at the threshold of being defined as DEGs. These findings reveal the complexity of such systems, in which, in mixed infections, one strain might benefit the other by potentiating its survival capabilities. Conversely, other types of mixtures might lead to intense competition between strains that, in combination with the host immune system response, may lead to the exclusion of one strain in the course of the infection. Nevertheless, our data suggest that during the early phase of *T. cruzi* infection, both strains coexist without interfering with one another.

In our experiments, it was improbable that many host cells were infected by both strains in the acute stage of the mixed infection, since we inoculated the mice with very low loads of parasites. Therefore, based on the parasitemia observed in the JG-infected and Col1.7G2-infected animals, we suggest that JG proliferates at a higher rate than Col1.7G2. This might explain why JG predominated in the hearts of double-infected BALB/c mice in the chronic phase. This hypothesis is corroborated by previous reports showing that both JG and Col1.7G2 were detected in the hearts of dually infected BALB/c mice by low-stringency PCR in the acute phase of infection, but in the chronic stage (3–6 months after infection) there was a predominance of JG in the hearts of these animals (Andrade et al., 1999, 2002). Additionally, recent findings by Dias et al. (2017) demonstrated that Col1.7G2 is less efficient than JG in proliferating in the cardiomyocytes of neonate BALB/c mice in culture, which reinforces our hypothesis.

We aim to better understand the differences among the pathways and processes modulated by JG and Col1.7G2. Mitochondrial metabolism is downregulated by all strains, although this reduction is less pronounced in Col1.7G2-infected animals, and translation and protein processing pathways were drastically decreased in both JG-infected and mixture-infected mice, but no effect was observed in Col1.7G2-infected mice. These alterations may represent a strategy to increase the viability of the infection, causing the adaptation of the parasite to the intracellular environment. Intracellular pathogens are known to secrete effector proteins capable of interfering with cell metabolism and signal transduction pathways. In this sense, the inhibition of MAP kinases and the NFκB pathway are mechanisms that pathogenic bacteria can exploit to increase their chances of survival inside a host cell (Cornejo et al., 2017). Additionally, pathogenic bacteria have mechanisms for inhibiting host protein translation to increase the availability of amino acids in the cytosol and can act to augment the uptake of nutrients from the cell (Best and Abu Kwaik, 2019). The data obtained in this study indicates that an unique capability to inhibit translation is also present in some strains of *T. cruzi* and may be an important mechanism to allow the persistence of the parasite in a specific organ. It is worth noting that the upregulation of genes related to angiogenesis, glucose homeostasis and muscle cell differentiation were also only observed in the JG- and mixture-infected groups.

Our findings suggest that JG might promote a reduction in the oxidative metabolism of infected cardiac cells. In general, there is no clear consensus in literature regarding if *T. cruzi* infection causes a reduction or an increase in the expression of genes controlling the energy production of the cell. Previous studies showed similar downregulation of oxidative phosphorylation genes in cardiomyocytes infected by distinct *T. cruzi* strains (Garg et al., 2003; Vyatkina et al., 2004; Manque et al., 2011; Best and Abu Kwaik, 2019). Experiments *in vivo* using mouse models during early timepoints, are in consistency with our findings that these genes decrease expression over disease progression (Garg et al., 2003; Vyatkina et al., 2004). Also, studies with cell cultures of human and mouse cardiomyocytes have shown the increase in the expression of such energy metabolism pathways (Shivali et al., 2009; Libisch et al., 2018). Is important to note that such variations might be result of genetic variability of strains or the timing of the *T. cruzi* infection. The downregulation of electron transport chain and oxidative phosphorylation gene expression could either increase or decrease reactive oxygen species (ROS) levels, depending on the proton motive force, NADH/NAD + and CoQH_2_/CoQ ratios and O_2_ concentration and this is difficult to confirm *in vivo* (Murphy, 2009). However, previous studies have shown that ROS is a double-edged sword for *T. cruzi* parasites, as it acts as a signaling molecule for *T. cruzi* replication in macrophages at low concentrations (Paiva et al., 2012; Goes et al., 2016), but it can be harmful at higher concentrations (Aguiar et al., 2013). Interestingly, treatment with catalase reduces the multiplication of JG, but not Col1.7G2, in cardiomyocytes, suggesting that H_2_O_2_ acts as a signaling molecule during JG growth in these cells (Dias et al., 2017). Thus, we speculate that *T. cruzi* strains that are sensitive to ROS, such as JG, can have increased proliferation rates depending on the host and tissues involved in the acute phase and may thus display differential tissue tropism, as observed for the *T. cruzi* mixtures.

The enrichment analysis of the biological processes revealed a signature compatible with intense immune activation in Col1.7G2- and mixture-infected animals. Although JG-infected animals also presented a similar signature, we observed it to be much less pronounced. The upregulation of genes related to neutrophil chemotaxis, antigen presentation and inflammatory responses suggests that these transcripts originate from new cells arriving in the heart to manage *T. cruzi* infection. The increased expression of cytokines such as IFN-γ, IL-6, TNF, and IL-12 observed in Col1.7 G2-infected animals is in accordance with the previously described Th1 response observed in murine hosts that was induced by the Y and Colombian *T. cruzi* strains (Aliberti et al., 1996). However, JG-infected animals exhibit lower expression of the same genes. Similar to the CXC and CC chemokines involved in neutrophil and macrophage recruitment, transcriptional factors, such as the class II major histocompatibility complex transactivator (CIITA) and TBX21/TBET that are well-known Th1 cell-specific transcription factors, also showed lower expression levels in the JG-infected group compared to the Col1.7G2-infected group. Notably, animals inoculated with a mixture of both parasites generally exhibited the same pattern in terms of immune response DEGs as the Col1.7G2-infected group. This expression pattern suggests that the Col1.7G2 strain is more likely to be recognized earlier by the immune system and trigger a stronger immune response than the JG strain.

**FIGURE 7:**
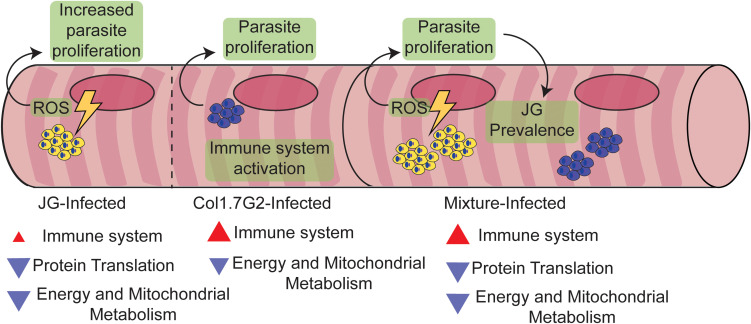
Proposed model explaining the preferential prevalence of JG compared to Col1.7G2 in BALB/c mouse hearts. Throughout the acute stage of *T. cruzi* infection, Col1.7G2 parasites induce a strong Th1-polarized immune response in mouse hearts. Although a potent Th1 response helps protect against parasites, amastigote nests are capable of surviving in later chronic stages. Mice infected with the JG strain exhibited a lower Th1 response and higher survival rates compared to mice infected with Col1.7G2. In addition, JG parasites, when stimulated by a higher sensitivity to ROS generated by the downmodulation of mitochondrial metabolism, produced a rapid increase in clonal burden. During a mixed infection with both strains, JG parasites can outcompete the Col1.7G2 population due to enhanced proliferation.

In the present work, we demonstrated the differential ability of the two *T. cruzi* strains (JG and Col1.7G2) to modulate gene expression in the hearts of BALB/c mice during the acute phase of infection. We also showed how a mixture of these *T. cruzi* strains affected host gene expression. We described two major distinct differences. The hearts of mice inoculated with JG or the mixture of both strains exhibited the downregulation of many genes involved in oxidative metabolism and translation compared to the uninfected controls. However, the hearts of mice inoculated with Col1.7G2 and the mixture of both strains showed the strong activation of genes involved in innate and adaptive immune responses compared to non-infected hearts. It is interesting to note that mice infected with a mixture of JG and Col1.7G2 followed the expression profile of the strain that caused alteration in comparison to the non-infected animals. Corroborating previous findings, we propose here that the remarkable differences between the two *T. cruzi* strains in their ability to persist in BALB/c hearts in the chronic stage of CD can be explained by events initiated during the acute phase, i.e., the higher intracellular proliferation rate of JG and its ability to slowly activate the immune response of the host in the acute phase of infection, which are in contrast to the effects of Col1.7G2, which strongly activates the host immune response and has a slower proliferation rate in cardiomyocytes. JG parasites would also benefit from the robust reduction in the overall energetic status of the cell and the increase in ROS production to become better established in tissue (Figure 7). We suggest here that the different features of each *T. cruzi* strain in ROS signaling, proliferation and immune system evasion could determine the survival of one strain over the other and its prevalence in host tissues. Altogether, our research highlights the need for a better understanding of the effect of *T. cruzi* polyparasitism and the uniqueness of each *T. cruzi* strain and its interaction with the host.

## Data Availability Statement

The datasets presented in this study can be found in online repositories. The names of the repository/repositories and accession number(s) can be found below: https://www.ncbi.nlm.nih.gov/geo/query/acc.cgi?acc=GSE132132.

## Ethics Statement

The animal study was reviewed and approved by the Institutional Committee for Animal Ethics of UFMG (CEUA-UFMG, license 64/12) approved all experimental procedures used in this study.

## Author Contributions

TC designed and performed the majority of the experimental procedures and analyses, generated the figures, and wrote the manuscript. MC assisted with the experimental study. DC and DS performed real-time PCR experiments and analyses. MB and NT assisted in bioinformatics analyses. SP, ET, and CM were involved in discussions on the study and contributed with expert insights. EC provided parasite strains and was engaged in experimental guidance. AM conceived the study, provided financial support, participated in its design, and was involved in discussions. GF conceived the study, provided financial support, wrote the manuscript, and contributed with experimental and bioinformatics analyses. All authors assisted with manuscript preparation, revision, and agreed with its submission.

## Conflict of Interest

The authors declare that the research was conducted in the absence of any commercial or financial relationships that could be construed as a potential conflict of interest.

## Footnotes

1 https://www.bioinformatics.babraham.ac.uk/projects/fastqc/

2 https://CRAN.R-project.org/package=factoextra

3 https://CRAN.R-project.org/package=FactoMineR

4 https://pachterlab.github.io/kallisto/

5 http://pachterlab.github.io/sleuth/

6 https://bioconductor.org/packages/ComplexHeatmap/

7 https://bioconductor.org/packages/org.Mm.eg.db/org.Mm.eg.db

8 https://bioconductor.org/packages/topGO/

9 http://apps.cytoscape.org/apps/cluego

## Supplementary Material

FIGURE S1 Overall workflow. All major bioinformatics steps performed in this work.

FIGURE S2 Number of *T. cruzi* mapped reads per sample. Bar graphic showing the number of *T. cruzi* detected reads in each sample.

FIGURE S3 Principal component analysis of all sequenced samples. Principal component analysis of all samples suggests that sample 8 of Col1.7G2 has high similarity with non-infected profile and due to low number of *T. cruzi* read detection it was excluded from subsequent analysis.

TABLE S1 RNA quality and library size. The sample concentration, volume, total mass, RIN score, 28S/18S ratio quality information as submitted for sequencing.

TABLE S2 Wald-test result table. Statistical result of gene expression comparisons as determined by pairwise wald-tests. Infected groups were compared against non-infected controls. Significant genes for downstream analysis were obtained by filtering the whole list of genes with log2 fold changes > 1 or <−1 and adjusted-*p*-values (*q*-values) smaller than 0.01.

TABLE S3 Venn diagram genes. List of all shared and exclusive genes in each experimental group that were used to generate the Venn diagram. Genes were detected after comparing experimental groups with controls using the Wald test. Selected genes had a Log2 fold change > 1 or <−1 and *q*-value of 0.01.

TABLE S4 Filtered dataset of genes considered differentially expressed. Complete list of differentially expressed genes detected by the Likelihood ratio test from all experimental groups after filtering the full dataset of detected genes according to a Log2 fold change > 1 or <−1 and *q*-value of 0.01.

TABLE S5 Complete Gene Ontology analysis dataset. Complete list of enriched GO terms detected by running the gene list obtained from each k-means cluster (Figure 3) through the Fisher elim algorithm as described in the TopGO package.

TABLE S6 Genes contained in each k-means cluster. Table containing the genes per cluster as represented in the heatmap (Figure 3).

TABLE S7 Differentially expressed genes per GO category. Table with a complete list of all DEGs representing each enriched GO category.

TABLE S8 Primers used in the qPCR validations.
