# Supplementary figures and images for: Differential Modulation of Mouse Heart Gene Expression by Infection With Two Trypanosoma cruzi Strains: A Transcriptome Analysis
Source: Front Genet. 2020 Sep 3;11:1031. doi: 10.3389/fgene.2020.01031 (PMC7495023; doi:10.3389/fgene.2020.01031)

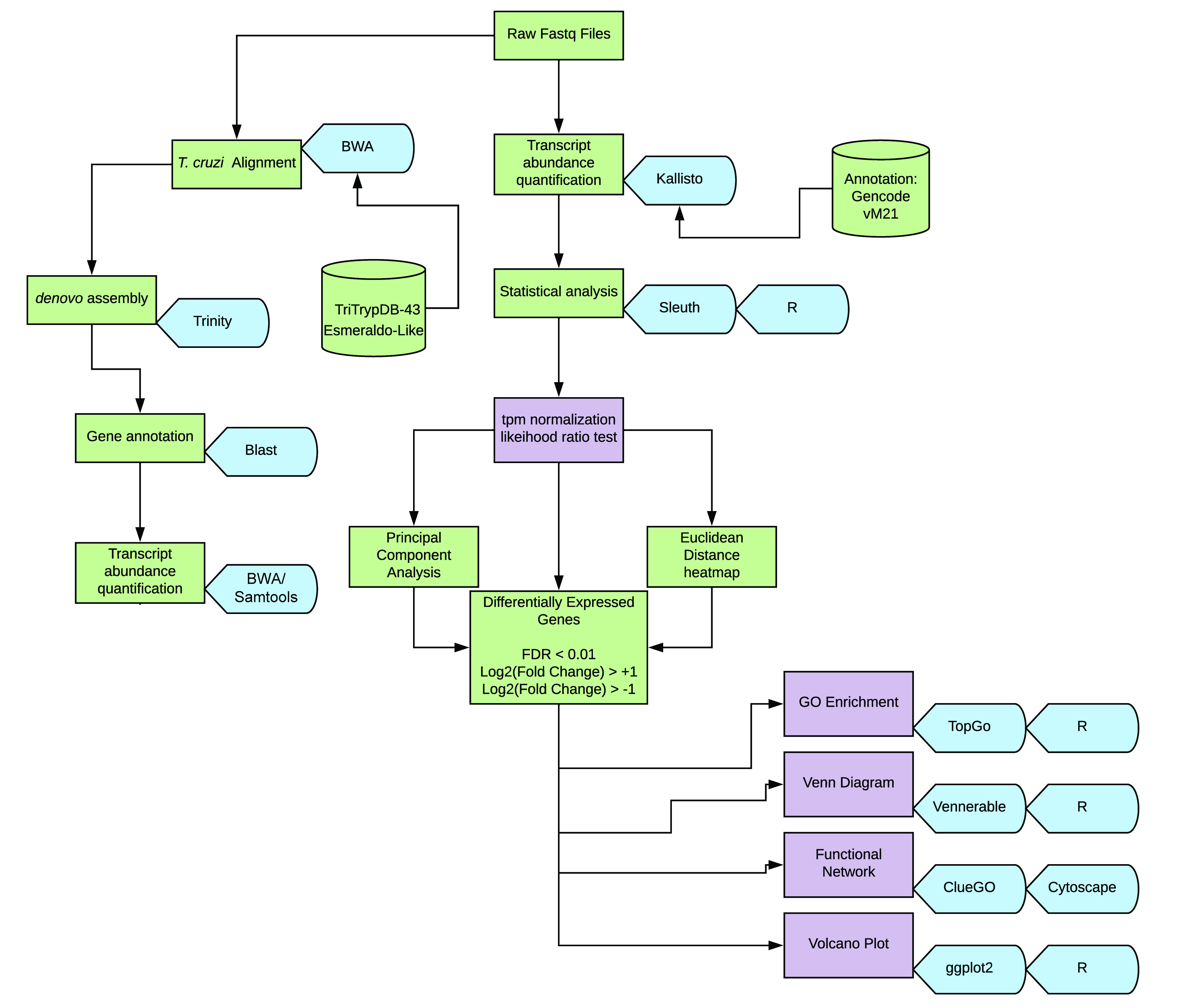

Supplement: FIGURE S1 — Overall workflow. All major bioinformatics steps performed in this work. [file Image_1.TIF]

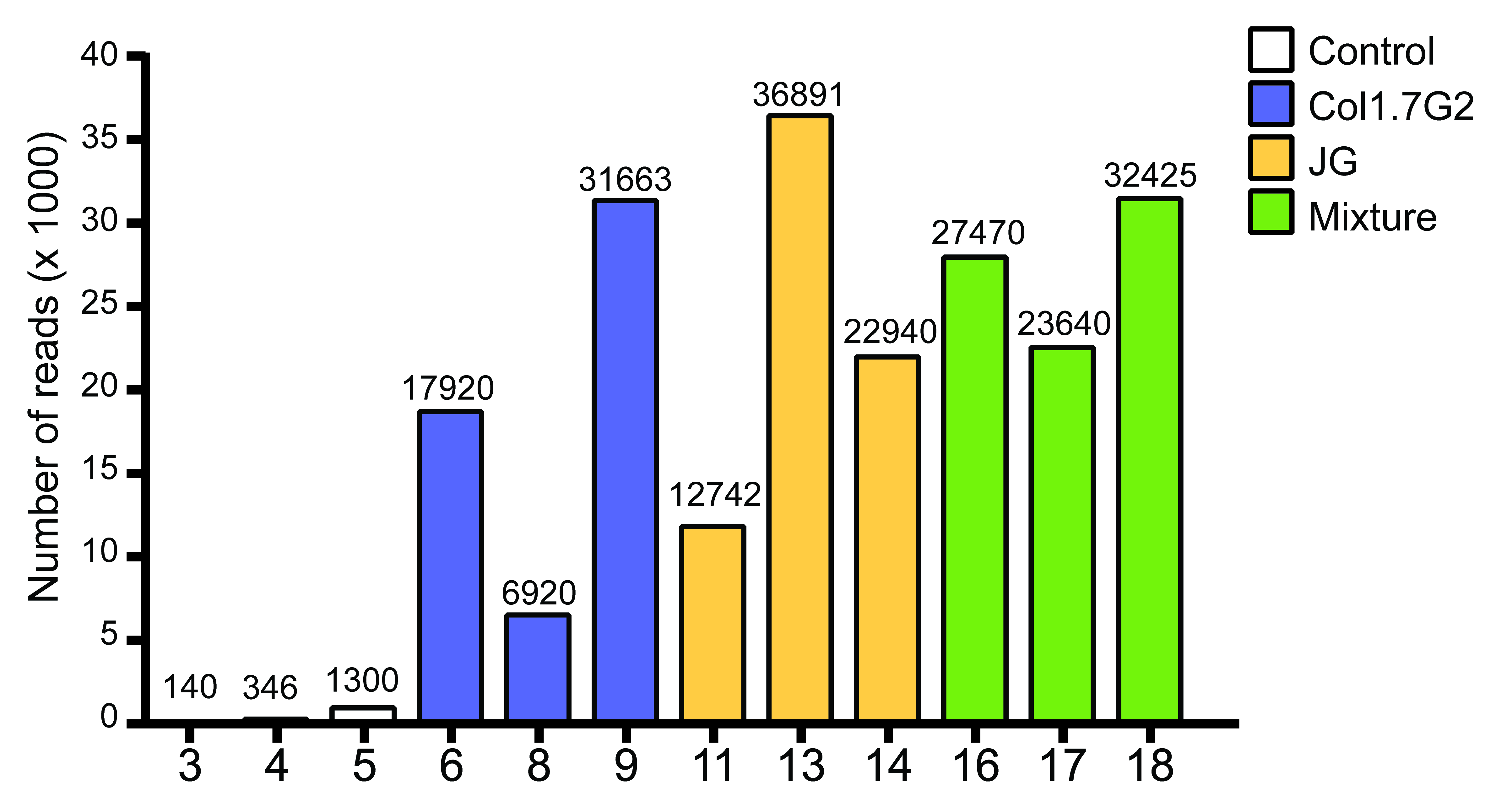

Supplement: FIGURE S2 — Number of T. cruzi mapped reads per sample. Bar graphic showing the number of T. cruzi detected reads in each sample. [file Image_2.TIF]

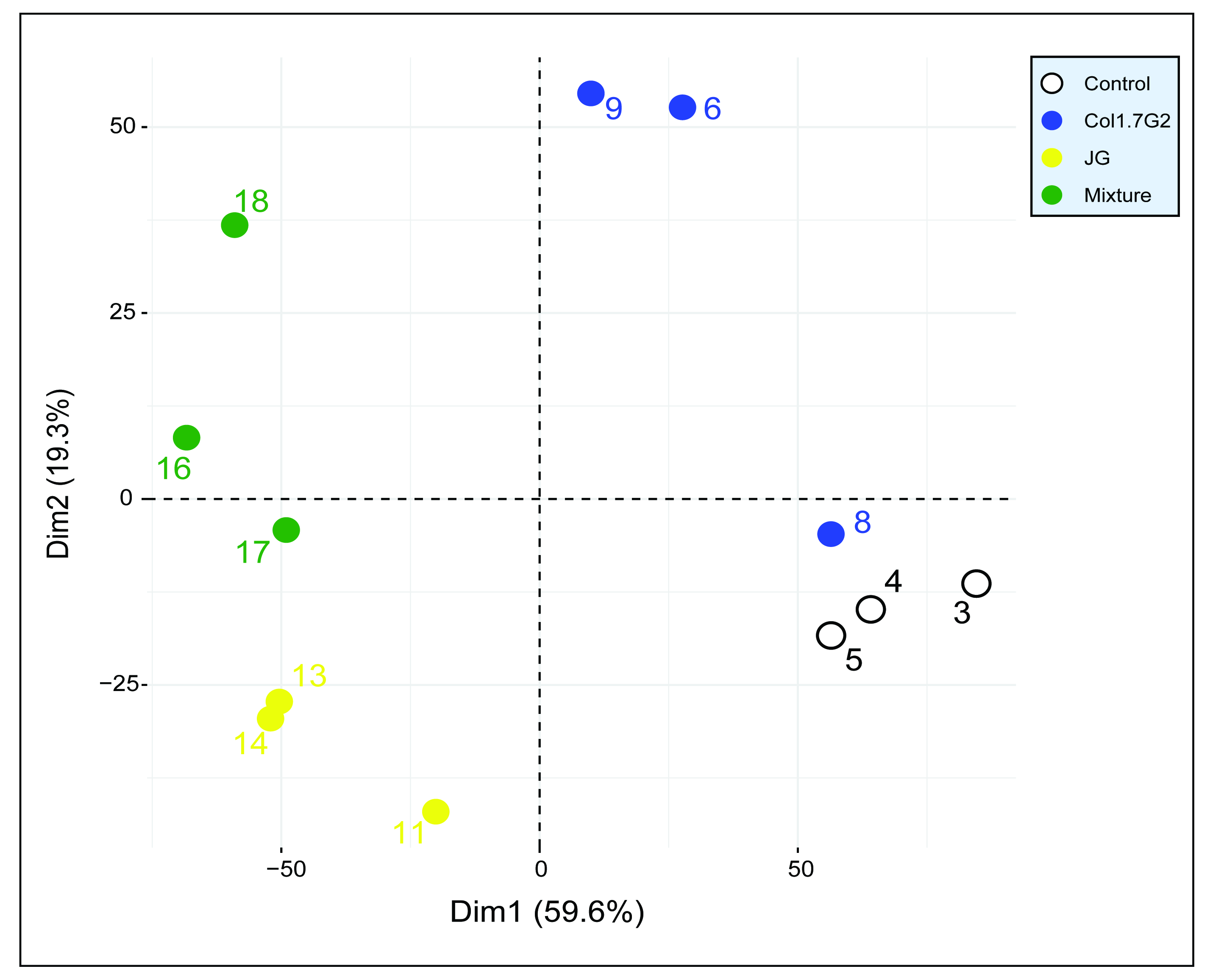

Supplement: FIGURE S3 — Principal component analysis of all sequenced samples. Principal component analysis of all samples suggests that sample 8 of Col1.7G2 has high similarity with non-infected profile and due to low number of T. cruzi read detection it was excluded from subsequent analysis. [file Image_3.tif]
